# An unusual suicidal death by automobile antenna: a case report

**DOI:** 10.1186/1746-1596-4-40

**Published:** 2009-11-24

**Authors:** Daniel C Lingamfelter, Erin Duddlesten, Reade A Quinton

**Affiliations:** 1 Jackson County Medical Examiners Office, Kansas City, Missouri, USA; 2 Southwestern Institute of Forensic Sciences, Dallas, Texas, USA; 3 University of Texas Southwestern Medical Center, and Medical Examiner, Southwestern Institute of Forensic Sciences, Dallas, Texas, USA

## Abstract

Worldwide, over one million people commit suicide every year. In the United States, the majority of these cases consist of gunshot wounds, hangings, and drug overdoses. Nevertheless, on rare occasions will individuals exhibit extraordinary creativity in how they decide to end their lives.

A decomposing white male was found lying beside his vehicle; blood was noted on the hood. Beneath the deceased's head was a copious amount of blood, and clutched within his right hand was a vehicle radio antenna. A search of a nearby storage shed uncovered drug paraphernalia and in the decedent's bedroom were two handwritten suicide notes. At autopsy, a defect was discovered in the right posterior oropharnyx. Immediately posterior to this injury were fractures of the right transverse processes and interarticular portions of the C2 and C3 vertebrae, with perforation of the right vertebral artery. No other injuries were noted externally. Radiographs of the head and neck showed no evidence of a projectile, and no corresponding exit defect was identified. Postmortem toxicology was positive for cocaine and methamphetamine.

We present a case report of a man under the influence of cocaine and methamphetamine employing a car antenna to cause self-inflicted, intraoral penetrating trauma to the cervical spine and right vertebral artery, resulting in exsanguination and his subsequent death.

## Background

Over one million individuals around the world end their own lives every year, translating into approximately one suicide every 40 seconds. These statistics have prompted the World Health Organization to proclaim September 10 "World Suicide Prevention Day" [1]. The most common methods of suicide encountered by forensic pathologists in the United States are of three principal methods - gunshot wounds, hangings, and overdoses - accounting for over 90% of suicides [2]. Regardless, some individuals can be rather creative in regard to how they kill themselves. We present a case report of a man under the influence of cocaine and methamphetamine employing a car antenna to cause self-inflicted, intraoral penetrating trauma to the cervical spine and right vertebral artery, resulting in exsanguination and his subsequent death.

## Case Presentation

### The Death Scene

**Figure 1:**
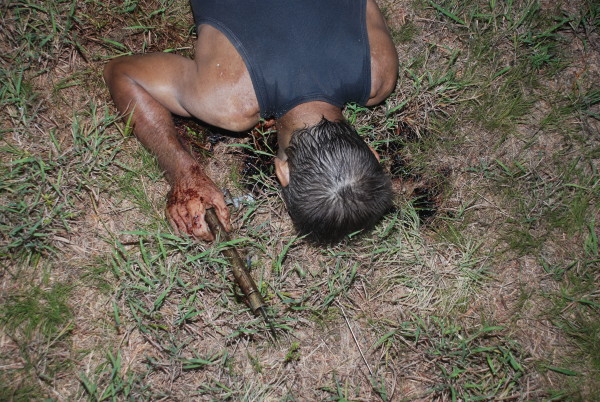
**The housing tube for a vehicle radio antenna was found grasped within the decedent's right hand**. Approximately two inches of antenna extended from the housing tube. This photograph additionally displays some of the blood found underneath the man's head.

A 43-year-old deceased white male was found lying prone in front of his automobile, facing away from the vehicle and wearing only a black tank top shirt and two white socks. He was last seen alive by his brother two days earlier. A large pool of dried blood and spattered blood lay over the car's hood. Due to recent heavy rains, some of this blood had run down the front of the vehicle, onto the bumper, and settled onto the ground beneath. A distinct circular indention was observed on the hood. Beneath the deceased's head was a copious amount of blood, and clutched within his extended right hand was a tubular metal object thickly covered in dried blood. Upon further inspection, it was discovered that this metal object was in fact the housing tube for a vehicle radio antenna, which would extend from the housing when the automobile is running and retract when the vehicle was powered off. Approximately two inches of antenna extended from the housing tube when it was found in the deceased's hand (Figure 1).

A search inside a metal storage shed in close proximity to the body uncovered assorted drug paraphernalia, including a metal spoon and empty syringe. It was confirmed through interviews with family members that the deceased abused illegal narcotics, including methamphetamine.

A search of the man's bedroom resulted in the recovery of a notebook containing two different handwritten and signed suicide-type notes, the first of which was an apparently compendious life statement, and the second an apology to his family. These notes had been written over two weeks prior to the discovery of the body.

### The Autopsy

**Figure 2:**
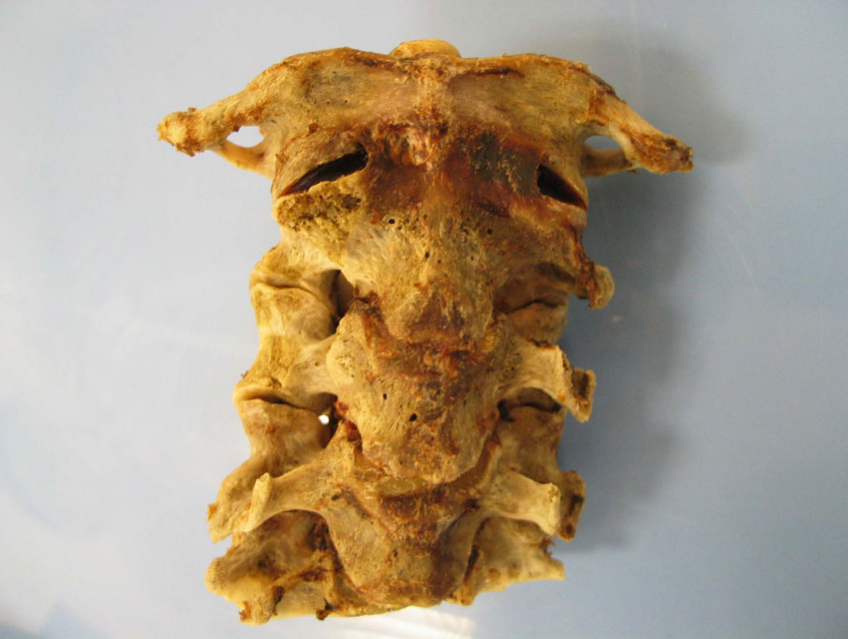
**(anterior view of cervical spine): The cervical spine was dissected from the body of the deceased, and the paraspinal tissues removed to display the injuries caused by the antenna - complete displacement of the right transverse processes and interarticular portions of the C2 and C3 vertebrae**.

The external examination was notable for moderate decompositional change and insect activity, concentrated over the head and chest. A 1/4 inch laceration was superior to the right eyebrow. No other injuries were noted externally. Inspection of the right posterior aspect of the oropharnyx revealed an irregular defect of the mucosa and soft tissues. Due to decompositional change, it was difficult to assess the presence of hemorrhage in the surrounding tissue. Immediately posterior to this injury were fractures and complete displacement of the right transverse processes and interarticular portions of the C2 and C3 vertebrae. Radiographs of the head and neck showed no evidence of a projectile, and no corresponding exit defect was identified. The right vertebral artery could not be visualized due to the degree of decomposition in this area of the body. However, given the fact that the foramina through which this vessel coursed were destroyed, it was surmised that the artery had been perforated. The cervical spine was removed for a more thorough examination of the bony injuries (Figure 2). The brain was negative for subarachnoid hemorrhage. The remainder of the autopsy was unremarkable.

Postmortem toxicology was positive for methamphetamine as well as for cocaine and cocaine metabolites.

Based upon the autopsy findings and the death scene investigation, the cause of death was ruled as "perforation of the right vertebral artery due to intraoral penetrating trauma with an automotive antenna." The manner of death was suicide.

## Discussion

This autopsy case represents an exceptional example of self-inflicted, intraoral penetrating vertebral artery trauma. Although a thorough literature search failed to uncover any exactly similar cases, a collection of incidences involving suicidal stab wounds to the throat have been reported [3,4], one of which involved vertebral artery trauma in a ten-year-old child who committed suicide by repeatedly stabbing himself in the throat [4]. Injuries included lacerations of the trachea, esophagus, and the right vertebral artery at the level of the C6 vertebral body. No mention of subarachnoid hemorrhage was provided.

When penetrating vascular injuries of the neck do occur, they are more often the result of motor vehicle accidents, falls, strangulation, and externally applied direct blows to the head and neck [5]. Other than penetrating trauma, vertebral artery damage has been shown to occur in a number of different fashions, oftentimes resulting from an overstretching of the vessel. These include chiropractic manipulation, rapid head rotation (for example, during yoga exercises or massage therapy), hyperextension, and hyperflexion [6]. It is within the third segment of the vertebral artery's course where it is most susceptible to stretching and subsequent trauma, since it is here that the vessel makes a tortuous lateral, then medial, trek from the C2 to C1 foramina. The case presented involved an injury of the right transverse processes of C2 and C3, within the second portion of the right vertebral artery. The trauma to this area reflects the unfortunate posterior, rightward location of this particular portion of the cervical spine in relation to the oropharnyx. Bony fixations of the vertebral artery to the C2 and C3 transverse foramina likely played a role in the vascular injury as a result of the fracture and displacement of these two transverse processes, with subsequent stretching and tearing of the artery from its housing.

The fact that there was no basilar subarachnoid hemorrhage (BSAH) in this case is not altogether surprising. One proposed mechanism for intracranial BSAH associated with extracranial vertebral artery rupture is that the injured artery must undergo dissection, and that blood ascends through the dissected layers of the vessel and eventually finds its way into the subarachnoid space. An alternative theory is that the blood tracks along nerve roots and enters the subarachnoid space of the spine, eventually making its way to the base of the brain [7]. Both theories only hold true if the vertebral artery is not completely transected, or if the injury is so close to the meninges to allow for direct sanguinous extension into the subarachnoid space. But, if the injury occurs in the more proximal first or second portions of the vessel, BSAH may be absent. Therefore, the absence of BSAH here can be justified by the relatively inferior location of injury, coupled with the severe foraminal bony destruction likely resulting in complete arterial perforation.

Based upon the copious amount of blood discovered at the death scene, an assumption can be made that much, if not most, of the blood spilling from the perforated artery escaped the body through the gaping posterior oropharyngeal defect created by the antenna, perhaps leaving little blood behind to result in significant internal hemorrhage.

It may be difficult to comprehend how anybody could inflict such violent, bizarre injury upon himself. A review of the deceased's postmortem toxicology revealed both acute cocaine and methamphetamine intoxication. Both of these illicit, sympathomimetic drugs are well established causes of paranoia, psychosis, depression, as well as violent, impulsive behavior sometimes culminating in states of excited delirium [8,9]. The National Violent Death Reporting System examined the toxicology profiles of suicide victims from thirteen states in the US and found that 9.4% of individuals tested positive for cocaine while 3.9% were positive for methamphetamine [10]. In several reports, methamphetamine specifically has been linked to unusually high suicide rates and suicide-completion rates [11,12]. It certainly does not take a stretch of one's imagination to theorize that the decedent, over two weeks after indicating that he was going to kill himself, finally harnessed enough stimulant-induced impulsivity and self-destructiveness to end his own life in quite gruesome fashion, especially considering the potential for pharmacologic synergism.

## Conclusion

This suicide represents an unusual example of self-destructive violence, as the decedent implemented a housing tube for a vehicle radio antenna to inflict penetrating trauma to the C2 and C3 vertebrae and subsequent perforation of the right vertebral artery.

The decompositional changes present during the autopsy created several challenges. First, the right vertebral artery could not be visualized in order to verify and document specific injuries to the vessel. Additionally, although the brain was still fairly well preserved to confidently rule out the presence of basilar subarachnoid hemorrhage, decomposition of the retropharnygeal structures obscured the presence of retropharyngeal blood.

Finally, the positive postmortem toxicology for two sympathomimetic agents raises the possibility that the intoxication of these substances led to the man's impulsive, self-directed violence, inflicted more than two weeks after he apparently had decided to end his own life.

## Competing interests

The authors declare that they have no competing interests.

## Authors' contributions

DL constructed the majority of the manuscript and was the forensic pathology fellow responsible for the autopsy case. ED provided technical autopsy assistance and was responsible for a significant portion of the manuscript. RQ was the staffing medical examiner for this autopsy case and was responsible for a significant portion of the manuscript. All authors read and approved the final manuscript.

## Consent

Written informed consent was obtained for publication of this case report and accompanying images. A copy of the written consent is available for review by the Editor-in-Chief of this journal.
